# How to quantify the evidence for the absence of a correlation

**DOI:** 10.3758/s13428-015-0593-0

**Published:** 2015-07-07

**Authors:** Eric-Jan Wagenmakers, Josine Verhagen, Alexander Ly

**Affiliations:** Department of Psychology, University of Amsterdam, Weesperplein 4, 1018 XA Amsterdam, The Netherlands

**Keywords:** Hypothesis test, Statistical evidence, Bayes factor

## Abstract

We present a suite of Bayes factor hypothesis tests that allow researchers to grade the decisiveness of the evidence that the data provide for the presence versus the absence of a correlation between two variables. For concreteness, we apply our methods to the recent work of Donnellan et al. (in press) who conducted nine replication studies with over 3,000 participants and failed to replicate the phenomenon that lonely people compensate for a lack of social warmth by taking warmer baths or showers. We show how the Bayes factor hypothesis test can quantify evidence in favor of the null hypothesis, and how the prior specification for the correlation coefficient can be used to define a broad range of tests that address complementary questions. Specifically, we show how the prior specification can be adjusted to create a two-sided test, a one-sided test, a sensitivity analysis, and a replication test.

After a Herculean effort involving a series of nine replication experiments, Donnellan et al. (in press) ultimately failed to reject the null hypothesis that people do not use warm showers and baths to compensate for a lack of social warmth, contradicting an earlier claim by Bargh and Shalev (2012). Unfortunately, the standard *p* value methodology does not allow one to quantify evidence in favor of the null hypothesis (Gallistel 2009; Rouder et al. 2009; Wagenmakers 2007). This is a major limitation, particularly for replication studies in which there is an important distinction between the statement “ *p*>.05, the data are uninformative” versus the statement “ *p*>.05, the data are informative and support the null hypothesis”.

It should be noted that the experiments from Donnellan et al. (in press) featured a total of 3073 participants; for such high-power experiments, one expects the outcome to be diagnostic, and hence it may be tempting to conclude that the non-significant *p* values reported by Donnellan et al. (in press) do indicate support in favor of the null hypothesis. However, this argument from power is insufficient, for two reasons. First, power is a pre-experimental expectation involving all possible outcomes, only one of which is relevant after the data are observed. In other words, even when conducting high-power experiments, researchers can be unlucky and obtain uninformative outcomes. To make this more concrete, consider an example featuring two urns (Wagenmakers et al. in press). One urn, $\mathcal {H}_{0}$, contains nine green balls and one blue ball. The other urn, $\mathcal {H}_{1}$, contains nine green balls and one orange ball. You are presented with one urn from which balls can be drawn with replacement, and your task is to determine the urn’s identity. Unbeknownst to you, the selected urn is $\mathcal {H}_{1}$. Your power analysis is based on the fact that a single draw has 10 % power, that is, $P(\text {reject } \mathcal {H}_{0} | \mathcal {H}_{1}) = P(\text {``draw orange ball''} | \mathcal {H}_{1}) = 0.10$. Consequently, an experiment with 90 % power requires that 22 balls are drawn (i.e., 1−0.9^22^). You carry out the experiment and you happen to draw 22 green balls: a completely uninformative result. This example demonstrates that high-power experiments need not provide diagnostic data. Second, even if the data could be argued to provide support in favor of the null hypothesis, the quantitative impact of this support remains unclear: are the observed data twice as likely under the null hypothesis $\mathcal {H}_{0}$ than under the alternative hypothesis $\mathcal {H}_{1}$, or 20 times, or perhaps 200 times?

Here we provide a series of Bayesian hypothesis tests to grade the decisiveness of the evidence that the data from Donnellan et al. (in press) provide in favor of the null hypothesis that people do not use warm showers and baths to compensate for a lack of social warmth. Throughout this article, we display a suite of Bayesian hypothesis tests: a default two-sided test for correlations (Jeffreys 1961), a default one-sided test for correlations (Boekel et al. in press), a sensitivity analysis, and a replication test for correlations (extending the work by Verhagen and Wagenmakers (2014)).

Our results show that although most *p* values from Donnellan et al. (in press) are non-significant, the evidence in favor of $\mathcal {H}_{0}$—as quantified by the default two-sided Bayesian hypothesis test—differs widely across the nine replication attempts: for the least informative attempt, the observed data are only two times more likely under $\mathcal {H}_{0}$ than under $\mathcal {H}_{1}$; for the most informative attempt, the observed data are 17 times more likely under $\mathcal {H}_{0}$ than under $\mathcal {H}_{1}$. Overall, the combined data from studies 1–4 (i.e., near-exact replications) and studies 5–9 (i.e., exact replications) are 16 and about 30 times more times more likely under $\mathcal {H}_{0}$ than under $\mathcal {H}_{1}$, respectively.

The methods outlined here are general and they can therefore be used equally well in other research domains whenever one seeks to quantify evidence for the absence or presence of a correlation. The relevant R code is illustrated through online materials available on the Open Science Framework at https://osf.io/cabmf/.

## The Donnellan data

In their studies 1a and 1b, Bargh and Shalev (2012) found that loneliness—as measured by the UCLA Loneliness Scale—correlated positively with the “physical warmth index”, a composite variable based on self-reported average frequency, duration, and temperature of showers and baths (*N* = 51, *r* = .57, *p*<.0001; *N* = 41, *r* = .37, *p*<.017). Based in part on these results, Bargh & Shalev (2012, p. 155) hypothesized that people “self-regulate their feelings of social warmth (connectedness to others) with applications of physical warmth (through taking warm baths or showers)”.

In this article, we reanalyze the data from the nine replication experiments conducted by Donnellan et al. (in press). As explained by Donnellan et al. (in press), studies 1–4 were near-exact replications (e.g., using a UCLA Loneliness Scale slightly different from the one used by Bargh and Shalev (2012)) and studies 5–9 were exact replications. In all nine studies, the focus of our analysis is the statistical association between loneliness and the physical warmth index used by Bargh and Shalev (2012).

**Fig. 1:**
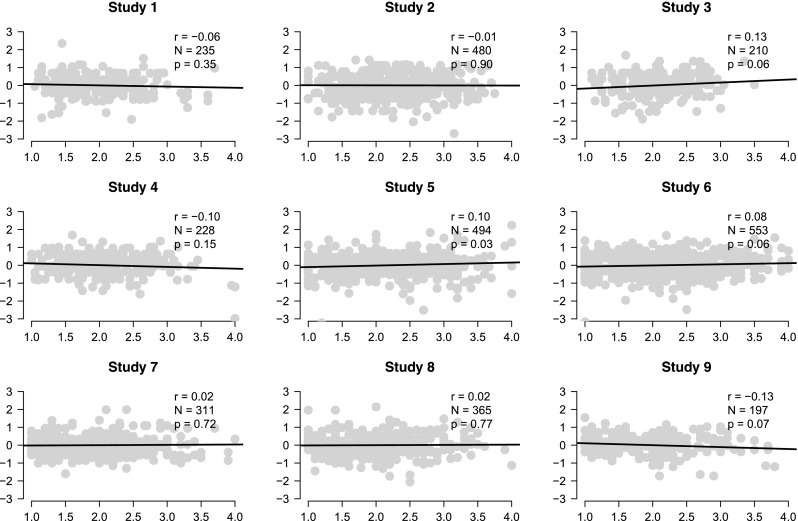
Data for the nine replication experiments from Donnellan et al. (in press). Scores for the loneliness scale are on the *x*-axis and scores for the physical warmth index are on the *y*-axis. Each panel also shows the sample Pearson correlation coefficient *r*, the number of observations *N*, and the two-sided *p* value

The first step in analyzing correlations is to plot the data and confirm that the assumption of a linear relation is appropriate (Anscombe 1973). For instance, a zero correlation between loneliness and the physical warmth index is misleading when the empirical relation is U-shaped. Figure 1 shows the raw data and confirms the validity of a standard correlational analysis. Across the nine experiments, the sample Pearson correlation values range from −.13 to +.13, and the associated two-sided *p* values range from .03 to .77.

## Posterior distributions

To quantify the evidence that the data provide for the presence and absence of a correlation *ρ* between loneliness and the physical warmth index, we need to contrast two statistical models: the null hypothesis $\mathcal {H}_{0}: \rho = 0$ and the alternative hypothesis $\mathcal {H}_{1}: \rho \neq 0$. In Bayesian inference, the complete specification of a statistical model requires that its parameters be assigned prior distributions (Dienes 2008; Lee and Wagenmakers 2013; Lindley 2014). For the Pearson correlation, the data are assumed to come from a bivariate normal, and this means that the model has five parameters: parameters *μ*_*x*_ and ${\sigma _{x}^{2}}$ are the mean and variance of the first variable, *μ*_*y*_ and ${\sigma _{y}^{2}}$ are the mean and variance of the second variable, and *ρ* is the correlation (see Appendix for details).

We start the specification of $\mathcal {H}_{1}$ by assigning uninformative, widely spread-out prior distributions to parameters *μ*_*x*_, *μ*_*y*_, ${\sigma _{x}^{2}}$, and ${\sigma _{y}^{2}}$ (Jeffreys 1961; Lee and Wagenmakers 2013; Ly et al. 2015).^1^ This leaves the specification of the prior distribution for the parameter of interest, the correlation *ρ*. At first we follow Jeffreys (1961) and assign *ρ* a prior that is uniform from −1 to 1; this prior reflects the belief that each value for *ρ* is equally likely before seeing the data. Hence, the alternative hypothesis is specified as $\mathcal {H}_{1}: \rho \sim U(-1,1)$.

**Fig. 2:**
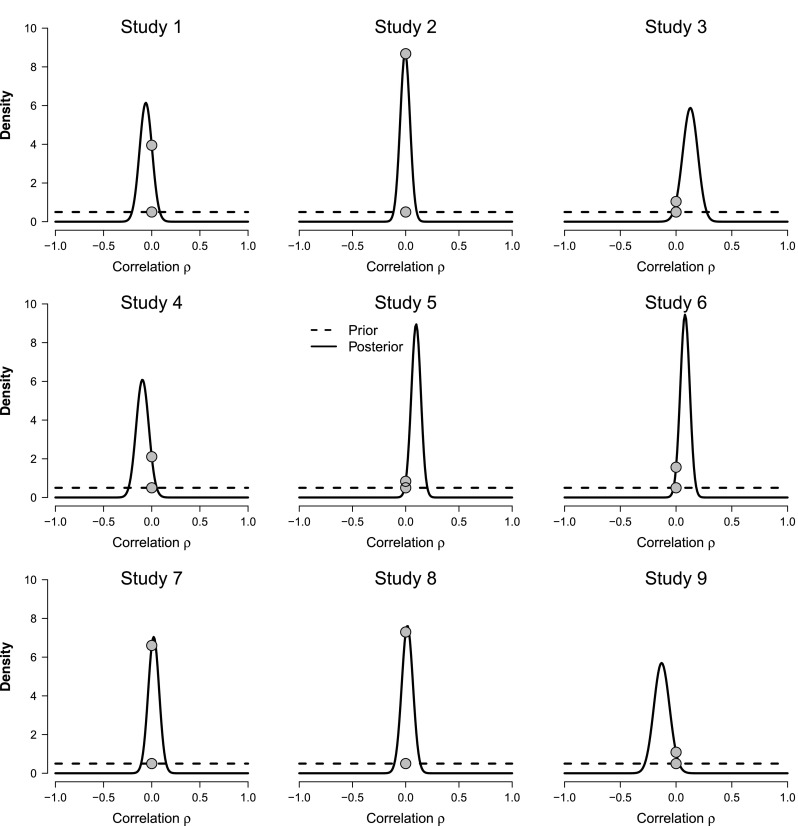
Prior and posterior distributions for the correlation *ρ* between loneliness and the physical warmth index across the nine replication experiments from Donnellan et al. (in press). The statistical model is defined as $\mathcal {H}_{1}: \rho \sim U(-1,1)$. The *filled dots* indicate the height of the prior and posterior distributions at *ρ* = 0; the ratio of these heights equals the evidence that the data provide for $\mathcal {H}_{1}$ versus $\mathcal {H}_{0}$ (Wagenmakers et al. 2010)

Assume for the moment that $\mathcal {H}_{1}$ is true and that we do not assign special status to the specific value *ρ* = 0; in that case our prior knowledge about *ρ* is completely captured by its prior distribution *ρ*∼*U*(−1,1). When data *d* arrive, this prior distribution *p*(*ρ*) is updated to a posterior distribution *p*(*ρ*∣*d*). The posterior distribution describes all that we know about *ρ* after seeing the data (and ignoring the fact that $\mathcal {H}_{1}$ may be false and *ρ* = 0 may deserve special consideration). To provide an initial intuitive impression about what the Donnellan data tell us about the correlation between loneliness and the physical warmth index, Fig. 2 shows prior and posterior distributions separately for each of the nine experiments.^2^

As is evident from each panel in Fig. 2, the data are informative in the sense that there is a substantial difference between the prior distribution and the posterior distribution. For studies 2, 7, and 8, the posterior distribution is approximately centered on *ρ* = 0; for studies 1, 4, and 9, most of the posterior distribution is concentrated on negative values of *ρ*; and for studies 3, 5, 6, most of the posterior distribution is concentrated on positive values of *ρ*. Although useful, a visual impression of the posterior distribution alone cannot serve to quantify the evidence that the data provide for the hypothesis that the correlation is present or absent, a topic we turn to next.

## Default Bayes factors

The Bayesian model selection and hypothesis testing machinery works as follows (Jeffreys 1961). Assume for simplicity that there are only two models under consideration, $\mathcal {H}_{0}: \rho = 0$ and $\mathcal {H}_{1}: \rho \sim U(-1,1)$. We start by assigning complementary prior probabilities to both hypotheses, that is $P(\mathcal {H}_{0})$ and $P(\mathcal {H}_{1}) = 1-P(\mathcal {H}_{0})$. Dividing these probabilities yields the prior model odds. For instance, a proponent of the relation between loneliness and bathing habits may believe that $P(\mathcal {H}_{0}) = .10$; hence, the proponent’s prior model odds equal $P(\mathcal {H}_{0})/P(\mathcal {H}_{1}) = 1/9$. Hence, this proponent believes that the presence of a correlation between loneliness and bathing habits is a priori nine times more plausible than its absence.

Of course, the specification of prior model odds is subjective. In this case, a skeptic may well have prior odds equal to $P(\mathcal {H}_{0}) / P(\mathcal {H}_{1}) = .99/.01 = 99$, meaning that this skeptic believes that the absence of a correlation between loneliness and bathing habits is a priori 99 times more plausible than its presence. In sum, the prior model odds can be used to measure an individual’s initial enthusiasm or skepticism regarding the hypotheses at hand.

Bayesian hypothesis testing, however, does not depend on prior odds; instead, it concerns itself with the change in prior odds brought about by the data. When the data *d* arrive, the prior model odds are updated to posterior model odds. Mathematically, the updating process proceeds as follows:

1 $$ \underbrace{\frac{P(\mathcal{H}_{0} \mid d)}{P(\mathcal{H}_{1} \mid d)}}_{\text{Posterior odds}}=\underbrace{\frac{P(\mathcal{H}_{0})}{P(\mathcal{H}_{1})}}_{\text{Prior odds}} \times \,\, \underbrace{\frac{P(d \mid \mathcal{H}_{0})}{P(d \mid \mathcal{H}_{1})}}_{\text{Bayes factor}}. $$

The Bayesian hypothesis test centers on the Bayes factor BF_01_: the extent to which the data change one’s belief about the plausibility of the competing models (Jeffreys 1961; Kass and Raftery 1995; Lee and Wagenmakers 2013). Thus, although proponent and skeptic may differ on their prior model odds (and, consequently, on their posterior model odds), as long as they agree on the model specification $\mathcal {H}_{1}: \rho \sim U(-1,1)$ they will agree precisely on the extent to which the data have changed their initial opinion. For instance, when BF_01_ = 8.5 the observed data are 8.5 times more likely under $\mathcal {H}_{0}$ than under $\mathcal {H}_{1}$, and when BF_01_ = 0.2 the observed data are five times more likely under $\mathcal {H}_{1}$ than under $\mathcal {H}_{0}$. Equation 1 shows that BF_01_ = 1/BF_10_; because the data from Donnellan et al. (in press) generally support $\mathcal {H}_{0}$, we prefer to report BF_01_ throughout, as odds larger than one are easier to interpret.

Thus, in order to grade the decisiveness of the evidence in the nine studies by Donnellan et al. (in press) we need to compute the Bayes factor $\text {BF}_{01} = p(d \mid \mathcal {H}_{0}) / p(d \mid \mathcal {H}_{1})$. When $\mathcal {H}_{1}: \rho \sim U(-1,1)$, this Bayes factor can be obtained easily (Jeffreys, 1961; see also the Appendix). The BF_01_ column of Table 1 shows the result. As expected from considering the posterior distributions shown in Fig. 2, the evidence in favor of $\mathcal {H}_{0}$ is particularly high for studies 2 (i.e., BF_01_ = 17.36), 7 (i.e., BF_01_ = 13.21), and 8 (i.e., BF_01_ = 14.60); each of these studies alone requires that we adjust our beliefs about the presence of a correlation between loneliness and the physical warmth index by more than an order of magnitude.

**Table 1:** Results from different Bayes factor hypothesis tests for each of the nine experiments from Donnellan et al. (in press), as well as for the data collapsed over studies 1–4 and studies 5–9

	*N*	*r*	*p*	BF_01_	BF_0+_	BF_0r_(*r*_orig_ = .57)	BF_0r_(*r*_orig_ = .37)
Study 1	235	–0.06	0.35	7.90	22.59	**16825.57**	39.37
Study 2	480	–0.01	0.90	17.36	19.24	17679.82	**47.45**
Study 3	210	0.13	0.06	2.09	1.08	50.25	**1.15**
Study 4	228	–0.10	0.15	4.21	28.58	21904.40	**35.05**
Study 5	494	0.10	0.03	1.67	0.85	134.72	**1.32**
Study 6	553	0.08	0.06	3.13	1.61	398.01	**2.98**
Study 7	311	0.02	0.72	13.21	10.32	**4894.19**	23.76
Study 8	365	0.02	0.77	14.60	11.84	**7002.82**	28.75
Study 9	197	–0.13	0.07	2.17	30.86	**21755.50**	28.25
Study 1-4	1153	–0.03	0.31	16.17	52.21	49671.92	70.00
Study 5-9	1920	0.01	0.56	29.53	20.53	31021.07	70.36

Note: *N* is the total number of participants, *r* is the sample Pearson correlation coefficient between loneliness and the physical warmth index, *p* is the two-sided *p* value, BF_01_ is the two-sided default Bayes factor in favor of $\mathcal {H}_{0}$, BF_0+_ is the one-sided default Bayes factor in favor of $\mathcal {H}_{0}$, BF_0r_(.57) is the replication Bayes factor in favor of $\mathcal {H}_{0}$ based on study 1a from Bargh and Shalev (2012) (featuring undergraduate participants, as in studies 1, 7, 8, and 9), and BF_0r_(.37) is the replication Bayes factor in favor of $\mathcal {H}_{0}$ based on study 1b from Bargh and Shalev (2012) (featuring participants from community samples, as in studies 2–6)

To visualize the Bayes factor results, Fig. 2 uses filled dots to indicate the height of the prior distribution versus the height of the posterior distribution at *ρ* = 0, assuming $\mathcal {H}_{1}$ holds. An identity known as the Savage-Dickey density ratio test (e.g., Dickey and Lientz (1970) and Wagenmakers et al. (2010)) states that the ratio between these heights equals BF_01_. For instance, consider the study 2 panel in Fig. 2. For that study, the data increased the plausibility of the point *ρ* = 0 by a factor of 17.36, meaning that at *ρ* = 0 the posterior distribution is 17.36 times higher than the prior distribution. This height ratio—obtained by considering only the prior and posterior distributions under $\mathcal {H}_{1}$—is identical to the Bayes factor BF_01_ between $\mathcal {H}_{0}$ and $\mathcal {H}_{1}$.

In addition, the evidence for $\mathcal {H}_{0}$ is rather weak for those studies in which the effect is in the predicted direction and most of the posterior mass is concentrated on positive values of *ρ*. Specifically, the results from studies 3 (i.e., BF_01_ = 2.09), 5 (i.e., BF_01_ = 1.67), and 6 (i.e., BF_01_ = 3.13) do not necessitate a substantial adjustment of our beliefs about the presence of a correlation between loneliness and the physical warmth index, as can be confirmed by the relative closeness of the dots on the distributions in the corresponding panels of Fig. 2. Note, however, that even for these relatively uninformative studies the evidence favors $\mathcal {H}_{0}$, whereas the respective classical *p* values equal *p* = .06 (i.e., “marginally significant”), *p* = .03 (i.e., “significant, reject $\mathcal {H}_{0}$”), and *p* = .06 (i.e., “marginally significant”).^3^

Finally, consider the evidence for the studies in which the effect is in the opposite direction and most of the posterior mass is concentrated on negative values of *ρ*. The results for studies 1 (i.e., BF_01_ = 7.90), 4 (i.e., BF_01_ = 4.21), and 9 (i.e., BF_01_ = 2.17) yield somewhat more evidence for $\mathcal {H}_{0}$ than did studies 3, 5, and 6, but the overall impression is less compelling than one might expect. The main reason for this is that our current Bayes factor is two-sided such that positive correlations constitute just as much evidence against $\mathcal {H}_{0}$ as negative correlations. For this particular scenario, however, there are strong expectations about the direction of the effect, and this warrants the application of a one-sided test.

### The one-sided test

For the two-sided test discussed in the previous section, the alternative hypothesis was specified as $\mathcal {H}_{1}: \rho \sim U(-1,1)$. This model specification expresses the belief that every value of the correlation *ρ* is equally likely a priori. However, the hypothesis proposed by Bargh and Shalev (2012) and tested by Donnellan et al. (in press) is clearly directional: the assertion is that lonely people take showers and baths that are warmer, not colder.

Within the Bayesian framework, it is conceptually straightforward to account for the direction of the hypothesis. Specifically, for the one-sided test the prior mass is assigned only to positive values of *ρ* such that $\mathcal {H}_{+}: \rho \sim U(0,1)$. The computation of the associated one-sided Bayes factor BF_0+_ is provided in the Appendix (see also Boekel et al., in press; Morey and Wagenmakers, 2014). The BF_0+_ column of Table 1 shows the result.

A comparison between the two-sided Bayes factor BF_01_ and the one-sided Bayes factor BF_0+_ reveals three regularities (see Table 1). The first regularity is that for the three studies where the posterior distribution from Fig. 2 was approximately symmetrical around *ρ* = 0, the evidence in favor of $\mathcal {H}_{0}$ is virtually unaffected; study 2: BF_01_ = 17.36 vs. BF_0+_ = 19.24; study 7: BF_01_ = 13.21 vs. BF_0+_ = 10.32; study 8: BF_01_ = 14.60 vs. BF_0+_ = 11.84. In fact, when the posterior distribution is perfectly symmetrical around zero, the two Bayes factors are identical (Wagenmakers et al. 2010).

The second regularity is that for the studies where the effect was in the predicted direction, the evidence is now more favorable to $\mathcal {H}_{+}$ than it was to $\mathcal {H}_{1}$; study 3: BF_01_ = 2.09 vs. BF_0+_ = 1.08; study 5: BF_01_ = 1.67 vs. BF_0+_ = 0.85; study 6: BF_01_ = 3.13 vs. BF_0+_ = 1.61. Under the one-sided test, the data from these studies have become almost completely uninformative. The data from study 5 even favor $\mathcal {H}_{1}$, although the strength of this support is so small that it does not merit attention (i.e., the data are 1/0.85≈1.18 times more likely under $\mathcal {H}_{+}$ than under $\mathcal {H}_{0}$). Thus, when the effect goes in the predicted direction the one-sided test makes the alternative hypothesis look better, but not by much. In fact, for a symmetrical prior a sign-restriction cannot increase the Bayes factor in favor of the alternative hypothesis more than two-fold (Klugkist et al. 2005; Wagenmakers et al. 2010).

The third regularity is that for the studies where the effect was in the opposite direction, the evidence is much less favorable for $\mathcal {H}_{+}$ than it was for $\mathcal {H}_{1}$; study 1: BF_01_ = 7.90 vs. BF_0+_ = 22.59; study 4: BF_01_ = 4.21 vs. BF_0+_ = 28.58; study 9: BF_01_ = 2.17 vs. BF_0+_ = 30.86. This is then the major difference between specifying a two-sided alternative hypothesis $\mathcal {H}_{1}$ and a one-sided alternative hypothesis $\mathcal {H}_{+}$: when the effect goes in the direction opposite to the one that was predicted, the evidence greatly favors $\mathcal {H}_{0}$. This happens because the evidence quantified by the Bayes factor is relative: when the observed effect is negative, this may be unlikely under $\mathcal {H}_{0}$, but it is even less likely under a model $\mathcal {H}_{+}$ that stipulates the effect to be positive.

In sum, by changing the prior distribution on *ρ* we can implement a one-sided Bayes factor that quantifies the evidence that the data provide for a positive correlation between loneliness and the physical warmth index. This one-sided test is arguably a better reflection of the underlying directional hypothesis, which states that lonely people take warmer—but not colder—showers and baths. Application of the one-sided test showed that the out of the nine replication experiments by Donnellan et al. (in press), three were not very informative. The other six studies, however, provided highly diagnostic information, each separately requiring a shift in belief towards $\mathcal {H}_{0}$ of more than an order of magnitude.

### Sensitivity analysis

The comparison between the two-sided and the one-sided Bayes factor has highlighted how the prior distribution on *ρ* can be used to specify different alternative hypotheses; and when different hypotheses are put to the test, different results will (and should) emerge. A persistent concern, however, is that the presented Bayes factor may be delicately sensitive to the specification of the prior, and that by specifying the prior at will, researchers can obtain any desired result. This concern can be addressed in more than one way. The most general counterargument is that the prior is an integral part of the model specification process—yes, one can specify a highly implausible and idiosyncratic prior on *ρ* to obtain a nonsensical result, but the specification of the prior is subject to criticism just as the specification of a highly implausible and idiosyncratic model structure (e.g., an exponential distribution for response times). In other words, silly models (whether through silly priors or silly structure) will lead to silly conclusions, but in many situations is it obvious when a model is silly and when it is not.

A related counterargument is that for many models, researchers can depend on default priors that are suitable for a reference-style analysis. This analysis can be refined if more knowledge is available, as was demonstrated above: we started with a two-sided default prior $\mathcal {H}_{1}: \rho \sim U(-1,1)$ and then refined the prior to $\mathcal {H}_{+}: \rho \sim U(0,1)$. An extreme form of refinement will be demonstrated in the next section. There, the prior distribution for the Bayes factor analysis of the Donnellan et al. (in press) studies is provided by the posterior distribution obtained from the Bargh and Shalev (2012) studies.

In this section, we explore another counterargument, namely to take the critique and evaluate it explicitly by means of a sensitivity analysis (e.g., Wagenmakers et al., 2011). In such an analysis, one calculates Bayes factors for a wide range of plausible prior distributions. If the conclusions depend on the prior specification in an important way, such that different plausible priors lead to qualitatively different Bayes factors, then it should be acknowledged that the data do not allow an unambiguous conclusion. However, it may also happen that the conclusions are qualitatively robust across a wide range of prior distributions (e.g., Wagenmakers et al., 2011). In our experience, such robustness is the rule rather than the exception.

For consistency with the two-sided tests carried out by Bargh and Shalev (2012) and Donnellan et al. (in press), we return to the two-sided Bayes factor BF_01_ that compares $\mathcal {H}_{0}: \rho =0$ to $\mathcal {H}_{1}: \rho \sim U(-1,1)$. One proposal for a sensitivity analysis could define a set of models by smoothly decreasing the range of the uniform distribution on *ρ*, such that $\mathcal {H}_{1}: \rho \sim U(-c,c)$, with *c*∈(0,1). We prefer a similar but more elegant solution, where we first rescale the correlation to lie between 0 and 1, and then assign it a beta distribution. Hence, *ρ*^′^∼beta(*α*, *α*), and a measure of the spread of this distribution is *γ* = 1/*α*. We then transform the beta distribution back to the (−1,1) scale and calculate the Bayes factors as a function of *γ*. When *γ* = 1, this corresponds to a uniform prior on the correlation coefficient, as per our default analysis. When *γ* = 0, which happens when *α* grows very large, $\mathcal {H}_{1}$ becomes indistinguishable from $\mathcal {H}_{0}$ and consequently the Bayes factor is 1. Values of *γ* in between 0 and 1 define an continuous range of different alternative hypotheses that represent different beliefs about the extent to which large values for the correlation are plausible.

**Fig. 3:**
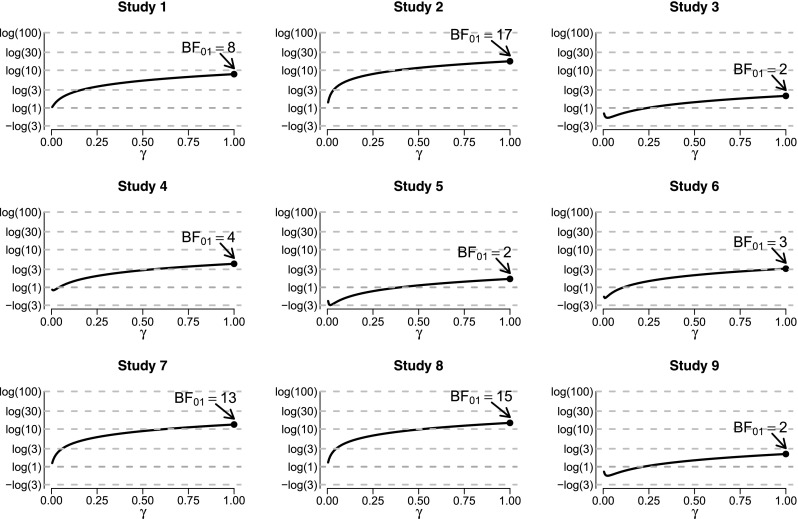
Sensitivity analysis for the Bayes factor BF_01_ across the nine replication experiments from Donnellan et al. (in press). The log of the Bayes factor BF_01_ is on the *x*-axis and the prior width *γ* is on the *y*-axis. When *γ* = 0 the alternative hypothesis equals the null hypothesis; when *γ* = 1 the alternative hypothesis is *ρ*∼*U*(−1,1). The Bayes factor is qualitatively robust in the sense that the evidence favors the null hypothesis across a wide range of prior beliefs. See text for details

Figure 3 shows the result of the sensitivity analysis for each of the nine experiments from Donnellan et al. (in press). The *y*-axis shows the log of the Bayes factor BF_01_, such that when *γ* = 0, all panels yield logBF_01_ = log(1)=0 and BF_01_ = 1, as predicted. In all panels, for all reasonable values of *γ*, the evidence supports the null hypothesis. In addition, there is no value of *γ* for which the evidence supports the alternative hypothesis in compelling fashion. Furthermore, for a large range of *γ* the Bayes factor does not show large fluctuations. Overall, the sensitivity analysis shows that, although different priors instantiate different models and will therefore yield different Bayes factors, it is not the case that any results whatsoever can be obtained. Instead, the qualitative results are similar across a range of plausible values for *γ*: the data provide clear evidence in favor of $\mathcal {H}_{0}$, but some experiments provide stronger evidence than others.

**Fig. 4:**
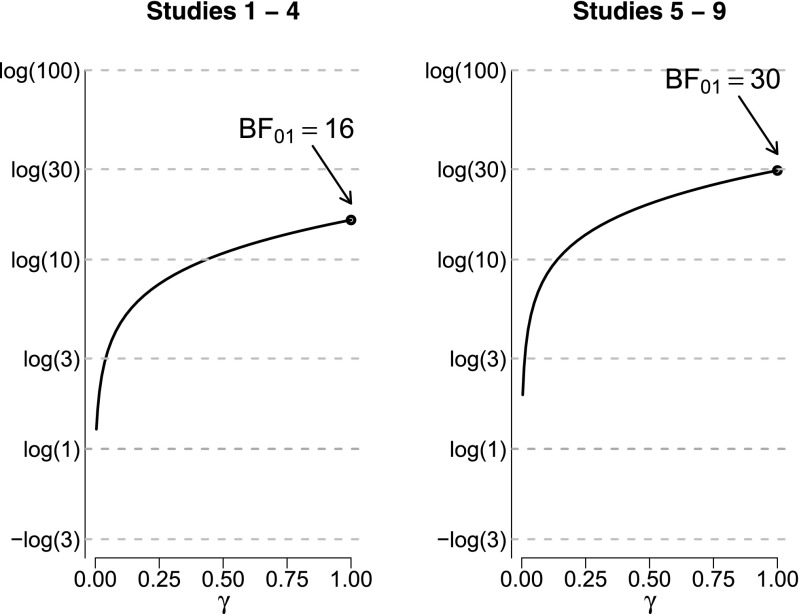
Sensitivity analysis for the Bayes factor BF_01_, collapsing data across studies 1–4 (*left panel*) and across studies 5–9 (*right panel*) from Donnellan et al. (in press). The log of the Bayes factor BF_01_ is on the *x*-axis and the prior width *γ* is on the *y*-axis. When *γ* = 0 the alternative hypothesis equals the null hypothesis; when *γ* = 1 the alternative hypothesis is *ρ*∼*U*(−1,1). See text for details

The same sensitivity analysis can be carried out after collapsing the data in two classes: one based on studies 1–4 and one based on studies 5–9. The studies within these two classes were highly similar (Donnellan et al. in press). Figure 4 shows the result. All values for *γ* result in Bayes factors that indicate support in favor of $\mathcal {H}_{0}$. When $\mathcal {H}_{1}$ is defined so as to predicts larger effects (i.e., through larger values of *γ*), the evidence more strongly supports $\mathcal {H}_{0}$. Thus, the more the models become distinguishable, the more the Bayes factor prefers $\mathcal {H}_{0}$.

It is insightful to compare the Bayes factors for the collapsed data from studies 1–4 (i.e., BF_01_ = 16.17) and studies 5–9 (i.e., BF_01_ = 29.53) to those obtained by multiplying the Bayes factors from the individual experiments. For studies 1–4, the multiplication yields 7.90×17.36×2.09×4.21≈1207; for studies 5–9, the multiplication yields 1.67×3.13×13.21×14.60×2.17≈2188. The discrepancy with the collapsed-data Bayes factors is large, and this serves to demonstrate that when effect sizes are related across studies—which is reasonable to assume– Bayes factors should not be multiplied (e.g., as was done by Bem et al. (2011) in order to present evidence in favor of extra-sensory perception). As explained by Jeffreys (1961, pp. 332–334), Bayes factors may only be multiplied when the prior distributions are properly updated. To clarify, consider two studies, *E*_1_ and *E*_2_, and a fixed effect. When the two experiments are analyzed simultaneously, the Bayes factor can be denoted BF(*E*_1_, *E*_2_), and it is obtained by integrating the likelihood over the prior distribution (see Appendix for details). When the two experiments are analyzed sequentially, the same end result should obtain, and this occurs with a Bayes factor multiplication rule based on the definition of conditional probability: BF(*E*_1_, *E*_2_)=BF(*E*_1_)×BF(*E*_2_∣*E*_1_). Note that the latter term is BF(*E*_2_∣*E*_1_), indicating that it is obtained by integrating the likelihood over the posterior distribution obtained after observing the first experiment. Thus, multiplying Bayes factors across *N* related units (participants or studies that show similar effects) is incorrect because the prior is used *N* times instead of being updated.

## Replication Bayes Factors

As outlined above, for replication studies there exists another way to alleviate the concern over how to specify the alternative hypothesis (Verhagen and Wagenmakers 2014). Specifically, one can use the data from the original study to obtain a posterior distribution, and then use that posterior distribution to specify the alternative hypothesis for the analysis of the replication studies. This “replication Bayes factor” therefore pits two models against one another. The first model, $\mathcal {H}_{0}: \rho = 0$, represents the belief of a skeptic, and the second model, $\mathcal {H}_{\text {r}}: \rho \sim \text {``posterior distribution from original study''}$, represents the idealized belief of a proponent. As pointed out by Verhagen and Wagenmakers (2014, p. 1459), “(...) the default test addresses the question, “Given that we know relatively little about the expected effect size beforehand, is the effect present or absent in the replication attempt?”; our test addresses the question, “Is the effect similar to what was found before, or is it absent?”. The two tests therefore represent extremes on a continuum of sensitivity to past research; the default test completely ignores the outcomes of an earlier experiment, whereas the replication test takes these outcomes fully into account.”

The replication Bayes factor was developed by Verhagen and Wagenmakers (2014) for the *t* test; here we extend that work to the Pearson correlation coefficient (for an application see Boekel et al. (in press); for mathematical details see the Appendix). Table 1 shows the results for two replication Bayes factors; the first, BF_0r_(.57), is based on study 1a from Bargh and Shalev (2012), featuring undergraduate participants and yielding *r*_orig_ = .57 with *n*_orig_ = 51; the second, BF_0r_(.37), is based on study 1b from Bargh and Shalev (2012), featuring a community sample of participants and yielding *r*_orig_ = .37 with *n*_orig_ = 41.

The BF_0r_(.57) column of Table 1 shows that, across all studies, the data are much more likely under the skeptic’s $\mathcal {H}_{0}$ than under the proponent’s $\mathcal {H}_{\text {r}}$ based on study 1a from Bargh and Shalev (2012). Even for the least compelling study, the data are 50.25 times more likely under $\mathcal {H}_{0}$ than under $\mathcal {H}_{\text {r}}$. When the proponent’s belief is based on study 1b from Bargh and Shalev (2012), the results are less extreme: the results for study 3 (BF_0r_(.37)=1.15), study 5 (BF_0r_(.37)=1.32), and study 6 (BF_0r_(.37)=2.98) are relatively uninformative: the data are almost as likely under the skeptic’s $\mathcal {H}_{0}$ than under the proponent’s $\mathcal {H}_{\text {r}}$. For the remaining studies, however, the results show compelling support for the skeptic’s $\mathcal {H}_{0}$, with Bayes factors ranging from about 23 to about 47.

**Fig. 5:**
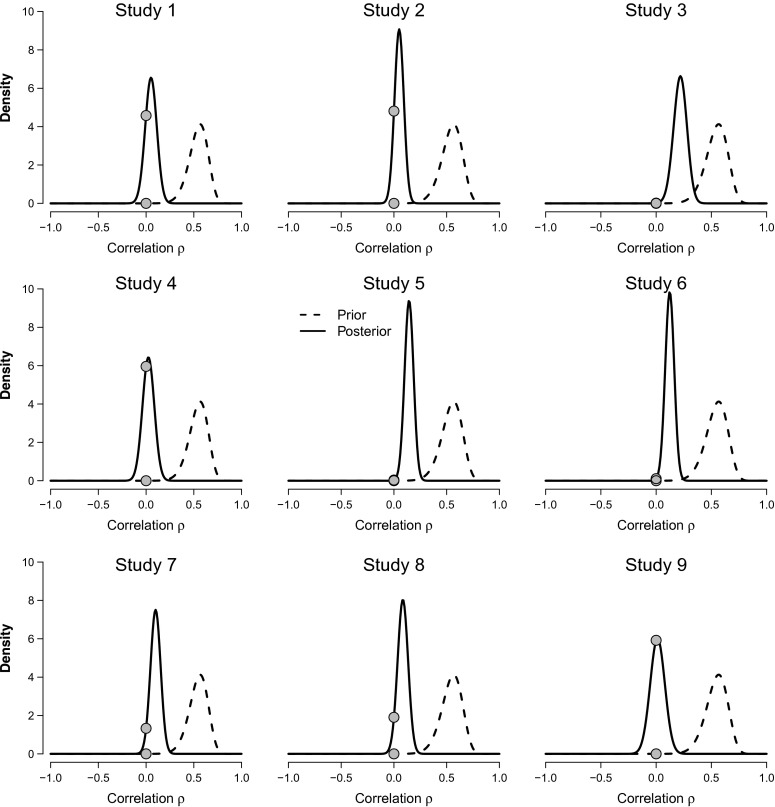
Prior and posterior distributions for the correlation *ρ* between loneliness and the physical warmth index across the nine replication experiments from Donnellan et al. (in press). The statistical model is defined as $\mathcal {H}_{\text {r}}: \rho \sim \text {``posterior distribution from original study''}$. The *filled dots* indicate the height of the prior and posterior distributions at *ρ* = 0; the ratio of these heights equals the evidence that the data provide for the proponent’s $\mathcal {H}_{\text {r}}$ versus the skeptic’s $\mathcal {H}_{0}$ (Wagenmakers et al. 2010)

Figure 5 visualizes the results using the Savage-Dickey density ratio. In each panel, the dotted line indicates the idealized belief of a proponent, that is, the posterior distribution from the original study by Bargh and Shalev (2012).^4^

As before, the Bayes factor BF_0r_ is given by the ratio of the height of the prior and posterior distribution at *ρ* = 0. For instance, the panel for study 1 shows that the value *ρ* = 0 is much more plausible after having seen the data from the replication study than before. In fact, the ratio of the prior and posterior density at *ρ* = 0 equals 16,825.57, which is equal to the replication Bayes factor BF_0r_(.57).

Similarly, the panel for study 3 shows that the data from the replication study have hardly altered the plausibility of the value *ρ* = 0 at all; hence the dot that indicates the height of the prior at *ρ* = 0 overlaps with the dot that indicates the height of the posterior at *ρ* = 0, and the replication Bayes factor equals BF_0r_(.37) = 1.15.

## Concluding comments

In this article we illustrated a suite of Bayesian hypothesis testing techniques that allow researchers to grade the decisiveness of the evidence that the data provide for the presence versus the absence of a correlation between two dependent variables. This approach is fundamentally different from Fisher’s *p* value methodology, which does not acknowledge the existence of an alternative hypothesis, and it is also fundamentally different from Neyman and Pearson’s frequentist tool for making decisions. As stated eloquently by Rozeboom (1960, pp. 422–423): “The null-hypothesis significance test treats ‘acceptance’ or ‘rejection’ of a hypothesis as though these were decisions one makes. But a hypothesis is not something, like a piece of pie offered for dessert, which can be accepted or rejected by a voluntary physical action. Acceptance or rejection of a hypothesis is a cognitive process, a degree of believing or disbelieving which, if rational, is not a matter of choice but determined solely by how likely it is, given the evidence, that the hypothesis is true.”

What, then, are the practical advantages of the Bayes factor hypothesis test over its classical counterpart? Among the most salient are the following: (1) Bayes factors allow researchers to claim evidence in favor of the null hypothesis (Gallistel 2009; Rouder et al. 2009; Wagenmakers 2007), an advantage that is particularly prominent in replication research such as that conducted by Donnellan et al. (in press); (2) Bayes factors allow researchers to quantify the above claim, so that we may know whether the data are more likely under $\mathcal {H}_{0}$ by a factor of 2, by a factor of 20, or by a factor of 200; (3) Bayes factors allow researchers to monitor the “evidential flow”^5^ as the data come in and stop data collection whenever this is deemed desirable, without the need for corrections depending on the intent with which the data were collected (Rouder 2014; Wagenmakers et al. 2012). This flexibility is a direct consequence of the Stopping Rule Principle (Berger and Wolpert 1988), a principle that all Bayesian analyses respect.

One may be tempted to argue that sensible conclusions can be reached using classical statistics when, in addition to the *p* value, the concept of power is taken into account. However, as alluded to in the introduction, power is a pre-experimental concept that entails averaging across all possible data sets, only one of which ends up being observed. It is therefore entirely possible that an uninformative result is obtained even after conducting a high-power experiment. For instance, consider studies 3, 5, and 6 from Donnellan et al. (in press); all our Bayes factor hypothesis tests indicated that these studies were virtually uninformative. Nevertheless, these studies featured 210, 494, and 553 participants, respectively. It is hard to argue that the uninformativeness of these data is due to a lack of power (see also Wagenmakers et al., in press).

Another tempting argument is that *p* values can quantify evidence for the null hypothesis when they are defined in a different manner. For instance, in equivalence testing (e.g., Rogers et al., 1993; Westlake, 1976) the null hypothesis is that an effect exists; when this hypothesis is rejected one accepts the hypothesis of equivalence. A similar method is to define a small effect, and use *p* values to test whether the observed effect is smaller than this small effect (Hodges and Lehmann 1954; Simonsohn, in press); the lower the *p* value, the more evidence there is in favor of the null hypothesis. Yet another method is based on confidence intervals; when confidence intervals are tightly centered on the value under scrutiny, this is felt to be evidence for the null hypothesis. These methods, however ingenious or intuitive, all suffer from two main limitations. First, they focus on a single hypothesis (for equivalence testing: the null hypothesis; for confidence intervals: the alternative hypothesis) and ignore what can be expected under the competing hypothesis. Second, they are unable to quantify evidence in a formal sense, and any evaluation of the end result remains to some extent based on an intuitive translation; consequently, these classical methods appear to be better suited for all-or-none decisions rather than for an assessment of the extent to which the data support one hypothesis over the other.

Some psychologists and statisticians object to hypothesis testing on the grounds that a point null hypothesis (e.g., *ρ* = 0 exactly) is known to be false from the outset (e.g., Cohen, 1994; Meehl, 1978). We disagree with this claim on principle (e.g., Iverson et al., 2010), but, more importantly, even if the claim were true it would not detract from the usefulness of hypothesis testing—instead, if could mean only that $\mathcal {H}_{0}$ needs to be specified with more care. For instance, for a test of the Pearson correlation coefficient one may replace $\mathcal {H}_{0}: \rho =0$ with $\mathcal {H}_{0}^{\prime }: \rho \sim U(-.01, .01)$. After specifying such an interval null hypothesis (Morey and Rouder 2011), the same methods outlined in this article may then be applied, with virtually identical results. That is, “(...) the assignment of a lump of prior probability to the simple hypothesis is strictly a mathematical convenience and not at all fundamental.” (Cornfield 1969, p. 637).

What fundamentally distinguishes Bayes factors from alternative methods, such as those that are based on confidence or credible intervals, is that Bayes factors assign separate prior plausibility to $\mathcal {H}_{0}$. This stems from the epistemic argument, originally put forward by Wrinch and Jeffreys, that such assignment is essential in order to be able to obtain inductive evidence in favor of an invariance or a general law (e.g.,Wrinch & Jeffreys 1919, 1921, 1923; see also Jeffreys, 1980; Ly et al., 2015; Rouder et al., 2009). In the present work, the emphasis was on the ability of the Bayes factor to quantify evidence in favor of an invariance; here, the absence of a correlation. However, the method can be used more generally, to quantify evidence for or against an invariance—the Bayes factor does not assign special status to either $\mathcal {H}_{0}$ or $\mathcal {H}_{1}$.

Throughout this article we have demonstrated that the prior distribution fulfills a pivotal and useful role in Bayes factor hypothesis testing. When the prior on the correlation coefficient is uniform from −1 to 1, we obtain Jeffreys’ default test (for alternative Bayes factor tests on the correlation coefficient, see Dienes, 2014, and Wetzels and Wagenmakers, 2012); when this prior excludes negative values, we obtain a one-sided version of Jeffreys’ test that respects the directional nature of the hypothesis at hand. The robustness of the conclusions to alternative, plausible specifications of the prior distribution can be assessed with a sensitivity analysis in which the shape of the prior is varied in systematic fashion. Finally, the prior distribution can be based entirely on earlier results, that is, on the posterior distribution from the original experiment. By changing the prior distribution, one changes the specification of $\mathcal {H}_{1}$, and thereby the outcome of the Bayes factor. This underscores the fact that the Bayes factor is a relative measure, as it compares the support for $\mathcal {H}_{0}$ versus a specific $\mathcal {H}_{1}$. We view our results as a vivid and concrete demonstration of what Jeffreys himself hoped his work would accomplish, namely that “(...) more attention will be paid to the precise statement of the alternatives involved in the questions asked. It is sometimes considered a paradox that the answer depends not only on the observations but on the question; it should be a platitude.” (Jeffreys 1961, p. x).

Finally, it should be acknowledged that, in many cases, the data pass the interocular traumatic test (i.e., when the data hit you right between the eyes; (Edwards et al. 1963)) and it does not matter whether one carries out a classical analysis, a Bayesian analysis, or no analysis at all. This argument loses some of its force, however, when the data appear to support the null hypothesis and an intuitive assessment of evidential strength becomes non-trivial. At any rate, one purpose of statistics is to make our intuitive judgement precise and quantitative. We hope that the methods outlined in this article will help contribute to that purpose.

## Appendix: Statistical details

### The likelihood

In Bargh and Shalev (2012), the Pearson’s correlation coefficient is used to measure the linear association between loneliness and the physical warmth index, which we denote by *X* and *Y*, respectively. The Pearson’s population correlation coefficient is defined as follows:

2 $$\begin{array}{@{}rcl@{}} \rho &=& { \text{Cov} (X, Y) \over \sigma_{x} \sigma_{y}} \text{ or equivalently }\\ \rho &=& E \left [ \left ({X-\mu_{x} \over \sigma_{x}} \right ) \left ({Y-\mu_{y} \over \sigma_{y}} \right ) \right ], \end{array} $$

where *X* is taken to be normally distributed with population mean *μ*_*x*_ and population standard deviation *σ*_*x*_. Similarly, *Y* is also assumed to be normally distributed such that *Y*∼(*μ*_*y*_, *σ**y*2). Assume that $\mathcal {H}_{1}$ holds and that there exists a correlation between *X* and *Y*. In order to describe the pair *X*, *Y* simultaneously, we then require five parameters, the normality parameters *μ*_*x*_, *μ*_*y*_, *σ*_*x*_, *σ*_*y*_ and the linear association *ρ*, resulting in the following likelihood:

3 $$\begin{array}{@{}rcl@{}} L(\mathcal{H}_{1} \, | \, d)& = & \left ({1 \over 2 \pi \sigma_{x} \sigma_{y} \sqrt{1- \rho^{2}}} \right )^{n} \\ && \times \exp \left( - {1 \over 2 (1-\rho^{2})} \sum\limits_{i=1}^{n} \left[ \frac{(x_{i}-\mu_{x})^{2}}{\sigma_{x}^{2}}\right.\right.\\&&\left.\left. + \frac{(y_{i}- \mu_{y})^{2}}{\sigma_{y}^{2}} + \frac{2 \rho (x_{i} - \mu_{x})(y_{i} - \mu_{y})}{\sigma_{x} \sigma_{y}} \right] \right), \end{array} $$

where we have written _1_ = (*μ*_*x*_, *μ*_*y*_, *σ*_*x*_, *σ*_*y*_, *ρ*) for the five parameters and *d* for the observed data with *d* = (*x*_1_*y*_1_),…,(*x*_*n*_*y*_*n*_) and (*x*_*i*_*y*_*i*_) being the reported loneliness and physical warmth index of participant *i*.

When the null hypothesis of no linear association between *X* and *Y* holds true, this means that *ρ* is fixed at zero. Consequently, this yields a model with only four free parameters, _0_ = (*μ*_*x*_, *μ*_*y*_, *σ*_*x*_, *σ*_*y*_). More precisely, the likelihood for the null model given the observations *d* then depends on the four parameters as follows:

4 $$\begin{array}{@{}rcl@{}} && L(\mathcal{H}_{0} \, | \, d) = \left ({1 \over 2 \pi \sigma_{x} \sigma_{y}} \right )^{n} \\&&\exp \left( - {1 \over 2} \sum\limits_{i=1}^{n} \left[\frac{(x_{i}-\mu_{x})^{2}}{\sigma_{x}^{2}} + \frac{(y_{i}- \mu_{y})^{2}}{\sigma_{y}^{2}}\right] \right). \end{array} $$

Note that Eq. 4 is simply Eq. 3 with *ρ* = 0.

### The Bayes factor test proposed by Sir Harold Jeffreys

To test whether the population correlation *ρ* is zero, we compare Eq. 4 to Eq. 3. Although the true values of the parameters are unknown, we can quantify our degree of belief about the true values by means of prior distributions. These prior distributions act as weighting functions for the likelihood. Sir Harold Jeffreys proposed that for $\mathcal {H}_{0}$, we weight the likelihood Eq. 4 with respect to the population means *μ*_*x*_ and *μ*_*y*_ proportional to 1, mathematically, *p*(*μ*_*x*_)∝1 and *p*(*μ*_*y*_)∝1. Furthermore, the standard deviations are weighted proportional to their inverse, that is, *p*(*σ*_*x*_)∝1/*σ*_*x*_ and *p*(*σ*_*y*_)∝1/*σ*_*y*_. These weighting functions then fully specify the marginal or average likelihood for _0_. To obtain the marginal likelihood for _1_ we use the same weighting functions for the common parameters and we weight the effects of *ρ* uniformly over (−1,1), that is, *p*(*ρ*) = 1/2. Hence, the two marginal likelihoods are given by the following integrals (i.e., averages):

5 $$\begin{array}{@{}rcl@{}} P(d \, | \, \mathcal{H}_{0}) &\stackrel{Eq.~4}{=}& \int \int \int \int L(\mu_{x}, \mu_{y}, \sigma_{x}, \sigma_{y} \, | \, d)\\\ &&\times1 \text{d} \mu_{x} 1 \text{d} \mu_{y} {1 \over \sigma_{x}} \text{d} \sigma_{x} {1 \over \sigma_{y}} \, \text{d} \sigma_{y}, \end{array} $$

6 $$\begin{array}{@{}rcl@{}} P(d \, | \, \mathcal{H}_{1}) &\stackrel{Eq.~3}{=}& \int \int \int \int \int L(\mu_{x}, \mu_{y}, \sigma_{x}, \sigma_{y}, \rho \, | \, d)\\&&\times 1 \text{d} \mu_{x} 1 \text{d} \mu_{y} {1 \over \sigma_{x}} \text{d} \sigma_{x} {1 \over \sigma_{y}} \text{d} \sigma_{y} {1 \over 2} \, \text{d} \rho. \end{array} $$

The ratio of these two marginal likelihoods yields the Bayes factor BF_01_ = *P*(*d* | _0_)*P*(*d* | _1_) that allows us to compare the two models as discussed in the main text.

The above equations suggest that we have to compute nine intensive integrals in order to obtain the Bayes factor. Fortunately, this is unnecessary, as Jeffreys (1961) showed that contributions due to the nuisance parameters *μ*_*x*_, *σ*_*x*_, *μ*_*y*_, *σ*_*y*_ are the same in the two models _0_,_1_ and, therefore, drop out of BF_01_ making the Bayes factor only dependent on *ρ* as

7 $$ \text{BF}_{01} = {1 / \text{BF}_{10}} \text{, where }\text{BF}_{10} = {1 \over 2} {\int}_{\!\!\!-1}^{1} {(1- \rho^{2})^{{n-1 \over 2}} \over (1-\rho r)^{2n-3 \over 2}} \, \text{d} \rho, $$

where *r* refers to the sample correlation *r* that is defined as:

8 $$ r = {{{\sum}_{i=1}^{n} (x_{i} - \bar{x}) (y_{i} - \bar{y}) } \over \sqrt{{\sum}_{i=1}^{n} (x_{i} - \bar{x})^{2} {\sum}_{i=1}^{n} (y_{i} - \bar{y})^{2}}}. $$

### The one-sided extension

It is straightforward to extend Jeffreys’ result to a one-sided test of the null hypothesis _0_ that *ρ* = 0 versus the directional restriction *ρ*>0, which we denote by _+_. The extension only requires us to change the uniform prior of *ρ* from on (−1,1) to a uniform prior on (0,1), which yields:

9 $$ \text{BF}_{0+} = {1 / \text{BF}_{+0}}, \text{where } \text{BF}_{+0} = {\int}_{\!\!\!0}^{1} {(1- \rho^{2})^{{n-1 \over 2}} \over (1-\rho r)^{2 n-3 \over 2}} \, \text{d} \rho. $$

The integrals Eqs. 7, 9 are evaluated explicitly by Eqs. 11 and 12 respectively with *α* = 1.

### Sensitivity analysis

Equation 7 shows how the Bayes factor BF_10_ depends on the choice of prior *p*(*ρ*), which was set to the uniform prior yielding *p*(*ρ*) = 1/2 for every *ρ* in (−1,1). To study how sensitive the Bayes factor is to this prior choice, we consider the uniform distribution as a member of the following class of priors that we refer to as the symmetric scaled beta distributions

10 $$ p(\rho \mid \alpha) = {2^{1-2 \alpha} \over \mathcal{B}(\alpha, \alpha)}\left( 1-\rho^{2}\right)^{\alpha -1}, $$

where (*α*, *α*) is a beta function. Each *α*>0 yields a candidate prior and we define *γ* = 1/*α* as a measure of the spread of this distribution. When *γ* = 1, this corresponds to a uniform prior on the correlation coefficient, as per our default analysis. When *γ* = 0, which happens when *a* grows very large, $\mathcal {H}_{1}$ becomes indistinguishable from $\mathcal {H}_{0}$ and consequently the Bayes factor is 1. Values of *γ* in between 0 and 1 define an continuous range of different alternative hypotheses that represent different beliefs about the extent to which large values for the correlation are plausible. The Bayes factor depending on *α* is then given by BF_01_(*α*) = 1/BF_10_(*α*) where

11 $$\begin{array}{@{}rcl@{}} \text{BF}_{10}(\alpha)&=&\frac{2^{1-2 \alpha} \sqrt{\pi}} {\mathcal{B}(\alpha, \alpha)} \frac{\Gamma \left( \tfrac{n -1 + 2 \alpha}{2} \right)}{\Gamma \left( \frac{n+ 2 \alpha}{2} \right)}\\&&\times {_{2} F_{1}} \left (\frac{2n - 3}{4}, \frac{2 n -1}{4} \, ; \, \frac{n+2 \alpha}{2} \, ; \, r^{2} \right ), \end{array} $$

and where _2_*F*_1_ denotes Gauss’ hypergeometric function (Oberhettinger 1972, section 15). Similarly, we get BF_0+_(*α*) = 1/BF_+0_(*α*), where

12 $$\begin{array}{@{}rcl@{}} \text{BF}_{+0}(\alpha)\!\!&=&\!\!\text{BF}_{10}(\alpha) + \frac{2^{1-2 \alpha} (2n-3) r}{(n-1+2 \alpha)\mathcal{B}(\alpha, \alpha)}\\&&\!\!\!\times \thickspace {_{3} F_{2}} \left( \!1,\! \frac{2n\! -\! 1}{4},\! \frac{2 n\! +\!1}{4}; \frac{3}{2}, \frac{n\,+\,2 \alpha\! +\!1}{2}; r^{2}\!\! \right ). \end{array} $$

### The replication Bayes factor

A replication Bayes factor (Verhagen and Wagenmakers 2014) answers the question: “Is the effect from the replication attempt comparable to what was found before, or is it absent?” The Bayes factor BF_0r_ extracts the evidence within the data from the replication study, *d*_rep_, and compares the null hypothesis of no effect, _0_:*ρ* = 0, against the alternative hypothesis _r_ that the correlation is equal to what is found in the original study.

As a prior on *ρ* for _r_, we use the posterior density *p*_orig_(*ρ*) conditioned on the original data. This density *p*_orig_(*ρ*) summarizes the finding of the original study (Jeffreys 1961, p. 175, equation 9) and simplifies to:

13 $$\begin{array}{@{}rcl@{}} p_{\text{orig}}(\rho)&=&p(\rho \mid d_{orig}) \propto \frac{(1-\rho^{2})^{\frac{n_{\text{orig}}-1}{2}}}{(1-\rho r_{\text{orig}})^{\frac{2n_{\text{orig}}-3}{2}}}\\&&\times~{_{2}F_{1}} \left( \frac{1}{2}, \frac{1}{2}; \frac{2 n_{\text{orig}} -1}{2} ; \frac{1}{2}+ \frac{1}{2} r_{\text{orig}} \rho \right), \end{array} $$

where *r*_orig_, *n*_orig_ are the sample correlation coefficient and sample size of the original study (see also the suggestion in Robert et al. (2009), and the integral shown in Gradshteyn and Ryzhik, 2007, Eq. 3.197.3, p. 317).

The replication Bayes factor BF_0r_ in favor of the null against a replication of the original result boils down to the ratio of the posterior divided by the prior *p*_orig_(*ρ*) at the point of interest, that is, *ρ* = 0 (e.g.; Wagenmakers et al., 2010). Hence,

14 $$\begin{array}{@{}rcl@{}} \text{BF}_{0 \text{r}} &= & {P(d_{\text{rep}} \, | \, \mathcal{H}_{0}) \over P(d_{\text{rep}} \, | \, \mathcal{H}_{\text{r}}) } \end{array} $$

15 $$\begin{array}{@{}rcl@{}} &= & { p_{\text{orig}}(\rho=0 \, | \, d_{\text{rep}} ) \over p_{\text{orig}}(\rho=0 )} \end{array} $$

where *p*_orig_(*ρ* | *d*_rep_) denotes the posterior given the data in the replication study, that is,

16 $$\begin{array}{@{}rcl@{}} p_{\text{orig}}(\rho \, | \, d_{\text{rep}} ) ={1 \over C} \frac{ (1-\rho^{2})^{{n_{\text{rep}}-1 \over 2}} } {(1-\rho r_{\text{rep}})^{2n_{\text{rep}}-3 \over 2}} p_{\text{orig}} (\rho), \end{array} $$

where *r*_rep_, *n*_rep_ refer to the sample correlation coefficient and sample size of the replication study respectively, and we have written *C* for the normalization constant

17 $$\begin{array}{@{}rcl@{}} C= {\int}_{-1}^{1} \frac{ (1-\rho^{2})^{{n_{\text{rep}}-1 \over 2}} } {(1-\rho r_{\text{rep}})^{2n_{\text{rep}}-3 \over 2}} p_{\text{orig}} (\rho) \text{d} \rho \end{array} $$

which can be computed by numerical integration.

The above equations are implemented in R code and available on the Open Science Framework at https://osf.io/cabmf/. Some of the functionality of this R code will also be available in the JASP 0.6 release (jasp-stats.org)

## Footnotes

1 For the correlation test outlined here, the prior distribution for these nuisance parameters have no impact on the Bayes factor (Jeffreys 1961; Ly et al. 2015).

2 A complete Bayesian analysis can update the posterior for *ρ* across experiments. Here, we wish to provide an indication of the informativeness of each experiment separately.

3 Results such as these illustrate the strong statement by Edwards (1965, p. 400): “Classical significance tests are violently biased against the null hypothesis.” The bias originates from the fact that *p* values only consider the extremeness of the data under $\mathcal {H}_{0}$ and disregard what is expected under $\mathcal {H}_{1}$ (e.g., Wagenmakers et al., in press).

4 In order to obtain the posterior distribution from the original experiment we still require a prior. However, even for relatively small data sets the shape of the posterior distribution is not much affected by the choice of prior distribution, as expressed by the adage “the data overwhelm the prior”. Studies 1, 7, 8, and 9 featured undergraduate participants, and hence the dotted lines in the corresponding panels are based on study 1a by Bargh and Shalev (2012) (i.e., *r*_orig_ = .57,*n*_orig_ = 51); in contrast, studies 2–6 featured community samples of participants, and hence the dotted lines are based on study 1b by Bargh and Shalev (2012) (i.e., *r*_orig_ = .37,*n*_orig_ = 41). In each panel, the solid line indicates the posterior distribution that was obtained after updating the beliefs based on the original study (i.e., the dotted distribution) with the data from the replication study.

5 To the best of our knowledge, this term was introduced in the blog of Eliezer Yudkowsky.
